# Graph‐Theoretical Approach for Predicting Physicochemical Properties of Stiff‐Person Syndrome Drugs

**DOI:** 10.1002/open.70239

**Published:** 2026-06-07

**Authors:** Jabbar Ali, Yasir Ali, Maher Ali Malik, Yilun Shang

**Affiliations:** ^1^ Department of Basic Sciences and Humanities College of Electrical and Mechanical Engineering National University of Sciences and Technology (NUST) Rawalpindi Pakistan; ^2^ Department of Computer Science National University of Modern Languages (NUML) Rawalpindi Campus Pakistan; ^3^ School of Computer Science Northumbria University Newcastle UK

**Keywords:** glutamic acid, gradient boosting, heat map, leave‐one‐out cross‐validation, M‐polynomials, quantitative structure–property relationship analysis, stiff‐person syndrome, topological indices

## Abstract

This work applies quantitative structure–property relationship analysis to examine molecular characteristics linked to stiffness person syndrome (SPS), a rare neurological condition marked by persistent muscle rigidity and spasmodic episodes. Topological indices such as Zagreb indices and their modification are employed to examine their correlations with key physicochemical properties. Linear, quadratic, exponential growth, and power curve fitting models are applied to identify the best correlations, focusing on the maximum F‐statistic and the coefficient of determination R^2^. Furthermore, heatmap‐based correlation analysis is performed to visually quantify the strength and direction of correlations between the calculated topological indices and selected physicochemical parameters. In addition to classical regression techniques, gradient boosting, a powerful ensemble machine learning method, is utilized to enhance predictive accuracy and evaluate the contribution of each descriptor toward property estimation. Additionally, M‐polynomials are calculated, and their corresponding graphs are plotted to assess the structural features of potential therapeutic agents. The findings offer significant insights into the role of graph‐theoretical methods in drug discovery, advancing the computational design of targeted treatments for SPS.

## Graphical abstract

Graph‐theoretical descriptors derived from M‐polynomials are integrated with quantitative structure–property relationship modeling and machine learning to uncover structure–property relationships of drugs used in stiff‐person syndrome. Strong correlations between degree‐based topological indices and key physicochemical properties are revealed, demonstrating how molecular connectivity drives predictive accuracy and offering a computationally efficient pathway for screening and optimizing therapeutic candidates.

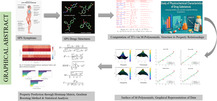

## 1 Introduction

Stiff‐person syndrome (SPS) [1] is a rare and complex neurological disorder that causes severe muscle stiffness, painful spasms, and difficulty with movement. It is an autoimmune disease. It happens when the body's immune system mistakenly attacks the nervous system, often targeting a protein called glutamic acid decarboxylase (GAD65), which plays a key role in calming muscle activity [2]. As a result, muscles become overly excitable, leading to uncontrollable rigidity and spasms. Individuals affected by this condition often suffer from repeated episodes of muscle rigidity in the limbs and trunk, along with spontaneous spasms triggered by increased stimulus sensitivity [3]. Approximately 65% of SPS patients lose the ability to manage daily tasks independently due to generalized stiffness, anxiety‐induced spasms, phobic reactions, and frequent falls. Some rely on mobility aids such as walkers or wheelchairs, while others become bedridden because of extreme rigidity. In SPS, substantial evidence indicates that disruption of γ‐aminobutyric acid (GABA)‐ergic signaling is driven by pathogenic autoantibodies; however, the precise antigenic target remains unidentified.

Muscle rigidity can occasionally be triggered by sudden loud sounds, such as car horns or other external stimuli. As a result, individuals may experience spasms and falls, leading to heightened fear of engaging in routine activities [4]. The symptoms of this disease are chronic pain, shortness of breath, exaggerated curve in the lower back, anxiety and agoraphobia, severe muscle spasms, muscle stiffness in the arms, legs, and torso, difficulty walking, unsteadiness, and falling [1]. Although the real cause is unknown, it may be due to an autoimmune reaction that attacks a protein that helps produce gamma‐aminobutyric acid (GABA). However, not all cases involve these specific antibodies, suggesting that other immune factors may be at play.

Managing SPS is challenging, as current treatments focus on relieving symptoms rather than stopping the disease itself. Medications like benzodiazepines (such as Diazepam and Clonazepam) and anticonvulsants (like Gabapentin and Pregabalin) can help relax muscles, but they don’t address the root cause. More advanced therapies [5], including intravenous immunoglobulin, plasmapheresis (a procedure that filters harmful antibodies from the blood), and B‐cell‐targeting drugs like Rituximab, are being explored to slow disease progression. However, there is still a pressing need for better treatments that not only ease symptoms but also tackle the immune system's role in SPS.

**FIGURE 1:**
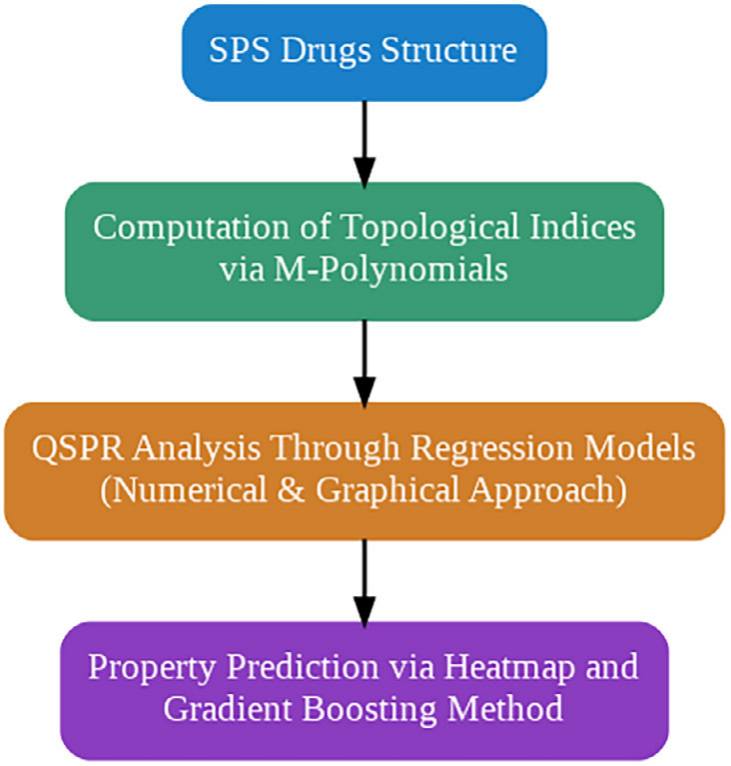
Workflow diagram for QSPR analysis of SPS drugs.

Developing these medicines and observing their results is an extremely time‐consuming method. It is very expensive and cannot be repeated multiple times with different medicines. This process is also very lengthy. To overcome such costly approaches, chemical graph theory and computational methods offer promising alternatives. In the past few years, computational methods have gained prominence in the field of drug discovery, particularly through the use of chemical graph theory and quantitative structure–property relationship (QSPR) modeling [6]. In recent developments within chemical graph theory and QSPR research, Imran et al*.* [7] employed M‐polynomial formulations to analyze biocontrol agents targeting Fusarium wilt, emphasizing the mathematical strength of algebraic graph invariants in representing molecular structures. Awan et al*.* [8] investigated degree‐based topological indices in relation to physicochemical attributes of antimalarial compounds, revealing significant correlations that validate the effectiveness of graph‐theoretic QSPR modeling. Complementarily, Shi et al*.* [9] reviewed emerging QSPR methodologies applied to cancer drug design, highlighting the integration of topological indices with modern machine learning frameworks for improved molecular characterization and prediction accuracy. Figure 1 shows a schematic for the QSPR analysis workflow.

Chemical graph theory utilizes molecular graphs where atoms are represented as nodes and bonds as edges. This approach enables the capture of structural features that influence molecular properties, providing a mathematical framework for analyzing molecular topology [10, 11]. Recent developments in QSPR modeling have emphasized the importance of incorporating diverse graph‐theoretical descriptors to enhance predictive performance. Degree‐based and modified reverse degree topological indices have been widely used to capture molecular connectivity and branching patterns, demonstrating strong correlations with physicochemical properties of drug molecules. Furthermore, neighborhood degree‐sum descriptors have been shown to provide improved structural characterization by incorporating local connectivity information, thereby enhancing model accuracy.

Topological indices, derived from these molecular graphs, are mathematical descriptors that capture key structural characteristics such as symmetry, connectivity, and branching [12]. These indices play a crucial role in correlating the molecular structure with various chemical and biological properties of compounds, providing valuable insights into their potential therapeutic effects. Recent trends in QSPR modeling have highlighted the effectiveness of machine learning techniques for the characterization of protein‐associated small molecules, particularly in small‐sample settings where descriptor selection and robust validation are critical for reliable prediction [13]. Recent advances have demonstrated the importance of graph‐theoretic coindices in characterizing molecular structures and supporting applications in drug design and targeted delivery systems [14].

In addition to connectivity‐based measures, distance‐based and degree–distance topological indices have been successfully applied to model complex molecular properties, particularly when integrated with regression and machine learning approaches. These advancements highlight the complementary role of different descriptor classes in QSPR analysis and motivate the use of multiple topological parameters for improved structure–property modeling.

Ali et al*.* conducted a recent study on potential therapeutic compounds for the treatment of Hantavirus pulmonary syndrome, employing leave‐one‐out cross‐validation (LOOCV) to ensure rigorous model validation and predictive reliability [15]. QSPR investigations extend far beyond hydrocarbons and nanotubes, finding applications in biochemistry and the study of biologically active compounds. For instance, Zaman et al*.* examines how molecular descriptors can be employed to predict both physical and biological characteristics of blood cancer drugs such as glasdegib, palbociclib, and daunorubicin. By leveraging degree‐based descriptors within QSPR frameworks, the study underscores their potential in guiding drug discovery and design [16]. Recent studies on applying Machine learning‐driven QSPR techniques have been proposed for the computational evaluation of drug candidates, where structure‐dependent topological indices are integrated with predictive models to predict key physicochemical properties [17]. Aqib et al*.* and Tchier et al*.* recently investigated QSPR analysis using reverse degree‐based topological descriptors incorporating drug discovery [18, 19].

QSPR, an advanced computational technique, is widely applied to relate topological indices, including topological indices, to a compound's physicochemical properties or biological activities [20]. In drug design, QSPR allows researchers to predict key pharmacokinetic parameters such as solubility, bioavailability, and permeability. By developing predictive models based on molecular structure, QSPR facilitates the optimization of drug candidates, enabling researchers to identify compounds that have a higher likelihood of success in treating diseases. This study applies QSPR modeling to investigate a group of drugs that are commonly used to treat symptoms of SPS: Diazepam, Clonazepam, Lorazepam, Pregabalin, Gabapentin, Tizanidine, Metronidazole, Carbamazepine, Valproate, and Ketamine [4]. These drugs vary in their chemical structure and mechanism of action, and their varying effectiveness in treating SPS presents an opportunity to investigate the relationship between their molecular properties and therapeutic outcomes.

To explore these relationships, several topological indices are calculated for the drugs under investigation [21]. These include the first Zagreb [22], second Zagreb, modified second Zagreb [23], redefined first Zagreb, redefined second Zagreb, redefined third Zagreb, forgotten index [24, 25], symmetric division degree, and augmented Zagreb index [26, 27]. The indices are derived using M‐polynomial techniques [28], which allow for a comprehensive representation of topological indices. These indices provide important insights into the structural characteristics of the compounds and serve as a basis for correlating their molecular structure to their therapeutic potential.

The **M‐polynomial** [28], originally proposed by Deutsch and Klavžar, serves as an important construct in chemical graph theory. It is widely utilized to derive closed‐form expressions for numerous degree‐based topological indices. For a graph *G*, the M‐polynomial is given by

(1) $$M(G;x,y)=\sum_{i\leq j}m_{ij}x^{i}y^{j}$$

where $m_{ij}=|\{uv\in E(G)|d_{u}=i,d_{v}=j\}|$, *E*(*G*) denotes the set of edges in *G*, *d*_u,_ and *d*_v_ represent the degrees of vertices u and v, respectively. In this formulation, *x* and *y* correspond to variables associated with vertex degrees, while *m* *_ij_* counts the edges connecting vertices of degrees *i* and *j*.

Alongside the topological indices, we also consider several physico‐chemical properties of the selected drugs [29], including molecular weight (MW), boiling point (BP), LogP (partition coefficient), molecular volume (MV), and density. These properties are essential for understanding the drug's absorption, distribution, metabolism, and excretion (ADME) profile. By evaluating these properties in conjunction with the topological indices, we can gain a deeper understanding of how the molecular structure of the drugs influences their overall pharmacokinetic behavior and their effectiveness in treating SPS. Although the present study does not focus on disease‐specific biological mechanisms, it provides a structural and physicochemical perspective on SPS‐related drugs, which may support future pharmacological investigations.

To analyze the data set, several curve‐fitting approaches are employed in MATLAB, including linear, quadratic, exponential growth, and power models. These fitting techniques help establish relationships between computed topological indices and the physicochemical attributes of the drugs. The performance and predictive reliability of these models are evaluated using statistical measures such as the correlation coefficient (*R*), coefficient of determination (*R* ^2^), root mean square error (RMSE), standard error, and the F‐statistic [30].

Additionally, M‐polynomial surface plots are generated to visually represent the relationships between the topological indices and physicochemical properties, allowing for a more intuitive understanding of how these topological indices contribute to the drugs’ effectiveness. Unlike conventional QSPR studies that primarily follow standard regression‐based workflows, this study incorporates an interpretable analysis using heatmap‐based correlation and a machine learning‐driven prediction framework, providing deeper insight into structure–property relationships. This approach not only helps identify the most significant molecular features influencing the therapeutic outcomes but also aids in visualizing the interactions between these features in the context of SPS treatment. Despite the number of QSPR models, SPS‐related drugs have not been systematically studied using graph‐theoretical descriptors within an interpretable and data‐driven framework. In this research, we combine topological indices with heat map‐based correlation analysis and a gradient boosting model to provide both interpretability and improved prediction of physicochemical properties. The rest of this paper is in the following pattern. Section 2 introduces the data set of SPS drugs, the molecular graph representation, and the computed topological indices. Section 3 presents the basic concepts underlying QSPR analysis, regression analysis, and statistical evaluation. Section 4 concludes the related literature and discusses the corresponding outcomes, respectively.

## 2 Materials and Methods

In this section, we provide a list of drugs commonly used for the treatment of SPS. The categorized list of drugs taken from the PubChem source [31] is given below:

• Diazepam ($C_{16}H_{13}{\mathrm{ClN}}_{2}O$),
• Clonazepam ($C_{15}H_{10}{\mathrm{ClN}}_{3}O_{3}$),
• Lorazepam ($C_{15}H_{10}{\mathrm{Cl}}_{2}N_{2}O_{2}$),
• Pregabalin ($C_{8}H_{17}{\mathrm{NO}}_{2}$),
• Gabapentin ($C_{9}H_{17}{\mathrm{NO}}_{2}$),
• Tizanidine ($C_{9}H_{8}{\mathrm{ClN}}_{5}S$),
• Metronidazole ($C_{6}H_{9}N_{3}O_{3}$),
• Valproate ($C_{8}H_{15}O_{2}$),
• Carbamazepine ($C_{15}H_{12}N_{2}O$),
• Ketamine ($C_{13}H_{16}\mathrm{ClNO}$)

All collected data were subjected to basic preprocessing, including unit standardization and consistency checks. Any missing or ambiguous entries were excluded from the analysis to avoid introducing bias. The finalized dataset was then used for subsequent QSPR modeling.

Using computational approaches to examine molecular structures, we derived multiple topological indices through vertex and edge partitioning methods, together with M‐polynomials [32, 7]. Topological Indices [12] were categorized into classical indices and modern descriptors. The complete list is shown in Table 1. A topological index is a mapping

**TABLE 1:** Computational formulas of topological indices.

Sr. No.	Topological Indices	Formula
1	First Zagreb [23]	$M_{1}=\sum_{\mathrm{uv}\in E(G)}(d_{u}+d_{v})$
2	Second Zagreb [23]	$M_{2}=\sum_{\mathrm{uv}\in E(G)}d_{u}d_{v}$
3	Modified Second Zagreb [23]	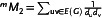
4	Augmented Zagreb [27]	$\mathrm{AZI}=\sum_{\mathrm{uv}\in E(G)}\;{\left(\frac{d_{u}d_{v}}{d_{u}+d_{v}}\right)}^{3}$
5	Redefined First Zagreb [24]	${\mathrm{ReZG}}_{1}=\sum_{\mathrm{uv}\in E(G)}\frac{d_{u}+d_{v}}{d_{u}d_{v}}$
6	Redefined Second Zagreb [25]	${\mathrm{ReZG}}_{2}=\sum_{\mathrm{uv}\in E(G)}\frac{d_{u}d_{v}}{d_{u}+d_{v}}$
7	Redefined Third Zagreb [25]	${\mathrm{ReZG}}_{3}=\sum_{\mathrm{uv}\in E(G)}(d_{u}d_{v})(d_{u}+d_{v})$
8	Hyper Zagreb [33]	$\mathrm{HZ}=\sum_{\mathrm{uv}\in E(G)}{\left(d_{u}+d_{v}\right)}^{2}$
9	Forgotten Index [25]	$F=\sum_{\mathrm{uv}\in E(G)}(d_{u}^{2}+d_{v}^{2})$
10	Symmetric Division Degree [34]	$\mathrm{SDD}=\sum_{\mathrm{uv}\in E(G)}\left(\frac{d_{u}}{d_{v}}+\frac{d_{v}}{d_{u}}\right)$

$$\begin{array}{c} \mathrm{TI}:G\to {\mathbb{R}}^{+} \end{array}$$

where *G* is a molecular (chemical) graph and $R^{+}$ denotes the set of positive real numbers. The value TI(G) represents the topological index corresponding to the structure of *G*. MATLAB software was used to compute the M‐polynomials and related topological indices for each drug, as summarized in Table 1.

Polynomial plots were generated for visualization, and the relationships between computed topological indices and different physicochemical properties were examined. Structural and physicochemical data for these drugs were obtained from PubChem [31], and ChemSpider [35]. Subsequently, QSPR analysis was carried out using the calculated indices, and statistical parameters were computed in MATLAB.

(2) Y=a+bX (Linearmodel)

(3) $$Y=a+bX+cX^{2}\;(\mathrm{Quadratic}\mathrm{model})$$

(4) $$Y=Ae^{bX}\;(\mathrm{Exponential}\mathrm{model})$$

(5) $$Y=AX^{b}\;(\mathrm{Power}‐\mathrm{law}\mathrm{model})$$

In this context, the topological index is represented by *X*, while *Y* denotes the corresponding physicochemical property. The regression coefficients are indicated by *a*, *b*, *c*, and *A*. The correlation coefficient is expressed as R, and Fisher's statistic as F. Model accuracy was assessed using RMSE and Standard Error (SE).

(6) $$R=\frac{\sum_{i=1}^{n}(y_{i}-\bar{y})({\hat{y}}_{i}-\bar{\hat{y}})}{\sqrt{\sum_{i=1}^{n}{\left(y_{i}-\bar{y}\right)}^{2}\sum_{i=1}^{n}{\left({\hat{y}}_{i}-\bar{\hat{y}}\right)}^{2}}}$$

(7) $$R^{2}=1-\frac{\sum_{i=1}^{n}{\left(y_{i}-{\hat{y}}_{i}\right)}^{2}}{\sum_{i=1}^{n}{\left(y_{i}-\bar{y}\right)}^{2}}$$

(8) $$\mathrm{RMSE}=\sqrt{\frac{1}{n}\sum_{i=1}^{n}{\left(y_{i}-{\hat{y}}_{i}\right)}^{2}}$$

(9) $$\mathrm{SE}=\sqrt{\frac{\sum_{i=1}^{n}{\left(y_{i}-{\hat{y}}_{i}\right)}^{2}}{n-p-1}}$$

(10) $$F=\frac{R^{2}/p}{\left(1-R^{2})/(n-p-1\right)}$$

In the equations above, $x_{i}$, ${\hat{x}}_{i}$, and σ denote the actual values, estimated values, and sample standard deviation, respectively. The notations are summarized in Table 2. We show the workflow for QSPR analysis of SPS drugs in Figure 1.

**TABLE 2:** Notation used in statistical performance metrics.

Symbol	Description
*y* *_i_*	Experimental (observed) value of the *i*th compound
${\hat{y}}_{i}$	Predicted value of the *i*th compound
$\bar{y}$	Mean of experimental values
$\bar{\hat{y}}$	Mean of predicted values
*n*	Total number of compounds in the dataset
*p*	Number of descriptors in the regression model

### 2.1 Formulas and Applications of the Drugs

Understanding the molecular formulas and pharmacological applications of the selected drugs provides insight into their structural behavior and biological activity. The chemical formula of a drug is directly linked to its therapeutic properties, influencing its interaction with biological targets. Such analysis is essential for the development of improved therapeutic agents and for guiding future research in medicinal chemistry and disease management.

Below, we summarize the fundamental characteristics and clinical applications of the drugs considered in this study

1. **Diazepam** (C_16_H_13_ClN_2_O): Widely used in the treatment of SPS, Diazepam reduces muscle spasms and rigidity by enhancing the inhibitory neurotransmitter gamma‐aminobutyric acid (GABA). Its action helps relieve the severe stiffness characteristic of SPS [36].
2. **Clonazepam** (C_15_H_10_ClN_3_O_3_): A benzodiazepine is prescribed for SPS to alleviate muscle rigidity and spasms. It enhances GABAergic activity, promoting muscle relaxation and improving neuromuscular control in affected individuals [37].
3. **Lorazepam** (C_15_H_10_Cl_2_N_2_O^2^): Used to manage muscle stiffness and spasms in neurological disorders. It enhances GABA‐mediated inhibition, thereby improving mobility and reducing discomfort [38].
4. **Pregabalin** (C_8_H_17_NO_2_): Commonly prescribed for neuropathic pain, epilepsy, and generalized anxiety disorder. It modulates voltage‐gated calcium channels, reducing neuronal excitability and pain transmission [39].
5. **Gabapentin** (C_9_H_17_NO_2_): Used for nerve pain, epilepsy, and restless leg syndrome. It acts on calcium channels to stabilize neuronal activity and reduce pain signaling.
6. **Tizanidine** (C^9^H_8_ClN_5_S): A centrally acting muscle relaxant used in multiple sclerosis and spinal cord injuries. It functions as an alpha‐2 adrenergic agonist, lowering muscle tone and alleviating spasticity.
7. **Metronidazole** (C_6_H_9_N_3_O_3_): An antibiotic and antiprotozoal agent used to treat anaerobic bacterial and parasitic infections. It disrupts microbial DNA synthesis, leading to cell death. Side effects may include nausea, dizziness, and a metallic taste.
8. **Valproate** (C_8_H_15_O_2_): An anticonvulsant and mood stabilizer used in epilepsy, bipolar disorder, and migraine prevention. It increases GABA levels in the brain, reducing neuronal excitability. Monitoring is required due to risks such as liver toxicity and teratogenicity.
9. **Carbamazepine** (C_15_H_12_N_2_O): An anticonvulsant and mood stabilizer prescribed for epilepsy, trigeminal neuralgia, and bipolar disorder. It stabilizes sodium channels to reduce abnormal neuronal firing. Regular blood tests are recommended to monitor potential hematological or hepatic effects.
10. **Ketamine** (C_13_H_16_ClNO): A dissociative anesthetic used for anesthesia, sedation, and pain management. It blocks NMDA receptors, producing analgesia and dissociation. At low doses, it is also used for treatment‐resistant depression and PTSD.

### 2.2 Molecular Structures of Drugs

**FIGURE 2:**
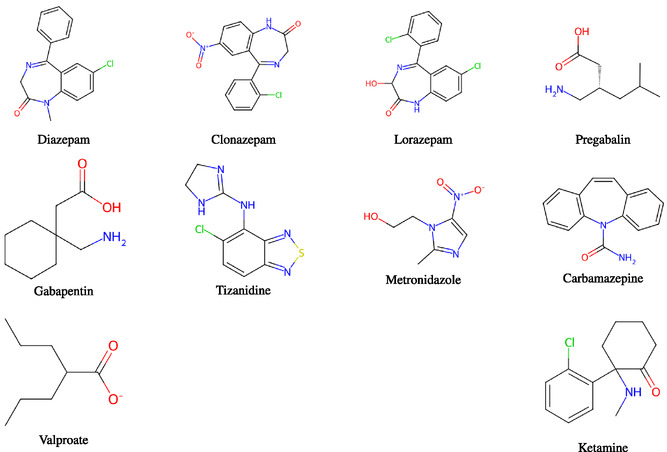
Molecular structures of the drugs (Source: PubChem https://pubchem.ncbi.nlm.nih.gov).

Every drug's molecular structure can be thought of as a graph, where atoms are connected by bonds, creating a unique arrangement. These molecular structures not only serve as the foundation for understanding various chemical descriptors but also offer deeper insights into their role in chemical and biological processes. Atoms such as carbon, hydrogen, oxygen, nitrogen, and sulfur are arranged in unique configurations to form each molecular structure. The structural representation of the drugs is provided in Figure 2.

The shape, size, and reactivity of a drug's molecular structure play a critical role in how it interacts with biological targets. For instance, fungicides, soil fumigants, and biological control agents are formulated to interact with specific targets, initiating biochemical processes that protect vegetables, legumes, and other crops from plant diseases.

The molecular configuration of a drug ultimately governs its physical and chemical characteristics, which in turn influence absorption, distribution, metabolism, excretion, and potential toxicity. These aspects collectively determine the drug's therapeutic effectiveness and overall pharmacological profile.

## 3 Computations of Topological Indices Using M‐Polynomials

Topological indices provide numerical invariants that describe structural characteristics of molecular graphs. By computing these indices for the selected drugs in Table 1, structural–activity correlations can be established.

The M‐polynomial is a unified tool for deriving several degree–based indices such as Zagreb indices, atom–bond connectivity, Forgotten index, and Sombor index. For a molecular graph with edge set *E*, let $E_{\left(d_{u},d_{v}\right)}$ represent the number of edges whose endpoints have degrees $d_{u}$ and $d_{v}$. For example, for Diazepam

$$\begin{array}{r} |E_{\left(1,3\right)}|=3,\;|E_{\left(2,2\right)}|=7,\;|E_{\left(3,3\right)}|=5,\;|E_{\left(2,3\right)}|=7 \end{array}$$

The M‐polynomials of the selected drugs are as follows:

1. **Diazepam** (C_16_H_1__3_ClN_2_O):
$$\begin{array}{r} M(G;x,y)=3xy^{3}+7x^{2}y^{2}+7x^{2}y^{3}+5x^{3}y^{3} \end{array}$$
2. **Clonazepam** (C_15_H_10_ClN_3_O_3_):
$$\begin{array}{r} M(G;x,y)=4xy^{3}+5x^{3}y^{3}+5x^{2}y^{2}+9x^{2}y^{3} \end{array}$$
3. **Lorazepam** (C_15_H_10_Cl_2_N_2_O_2_):
$$\begin{array}{r} M(G;x,y)=4xy^{3}+10x^{2}y^{3}+5x^{3}y^{3}+4x^{2}y^{2} \end{array}$$
4. **Pregabalin** (C_8_H_17_NO_2_):
$$\begin{array}{r} M(G;x,y)=4xy^{3}+5x^{2}y^{3}+xy^{2} \end{array}$$
5. **Gabapentin** (C_9_H_17_NO_2_):
$$\begin{array}{r} M(G;x,y)=xy^{2}+2xy^{3}+4x^{2}y^{2}+4x^{2}y^{4}+x^{2}y^{3} \end{array}$$
6. **Tizanidine** (C_9_H=ClN_5_S):
$$\begin{array}{r} M(G;x,y)=3x^{3}y^{3}+9x^{3}y^{2}+xy^{3}+5x^{2}y^{2} \end{array}$$
7. **Metronidazole** (C_6_H_9_N_3_O_3_):
$$\begin{array}{r} M(G;x,y)=3xy^{3}+2x^{2}y^{2}+3x^{2}y^{3}+xy^{2}+3x^{3}y^{3} \end{array}$$
8. **Carbamazepine** (C_15_H_12_N_2_O):
$$\begin{array}{r} M(G;x,y)=6x^{3}y^{2}+5x^{3}y^{3}+2xy^{3}+6x^{2}y^{2} \end{array}$$
9. **Valproate** (C_8_H_16_O_2_):
$$\begin{array}{r} M(G;x,y)=2xy^{2}+2x^{2}y^{3}+2xy^{3}+2x^{2}y^{2}+x^{3}y^{3} \end{array}$$
10. **Ketamine** (C_13_H_16_ClNO):
$$\begin{array}{r} M(G;x,y)=6x^{2}y^{2}+2xy^{3}+3x^{3}y^{3}+xy^{2}+5x^{2}y^{3} \end{array}$$

Using the definitions from Table 1, the computed indices for Diazepam are summarized below. Its M‐polynomial is:

$$\begin{array}{r} M(G;x,y)=3xy^{3}+7x^{2}y^{2}+7x^{2}y^{3}+5x^{3}y^{3} \end{array}$$

**First Zagreb index**: $M_{1}(G)=\left(12xy^{3}+28x^{2}y^{2}+35x^{2}y^{3}+30x^{3}y^{3}\right){|}_{x=y=1}=105$.

**Second Zagreb index**: $M_{2}(G)=\left(9xy^{3}+28x^{2}y^{2}+42x^{2}y^{3}+45x^{3}y^{3}\right){|}_{x=y=1}=124$.

**Modified second Zagreb index**: 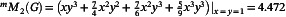.

**Redefined first Zagreb index**: ${\mathrm{ReZG}}_{1}(G)=\left(4xy^{3}+7x^{2}y^{2}+\frac{35}{6}x^{2}y^{3}+\frac{30}{9}x^{3}y^{3}\right){|}_{x=y=1}=20.1667$.

**Redefined second Zagreb index**: ${\mathrm{ReZG}}_{2}(G)=\left(\frac{9}{4}xy^{3}+7x^{2}y^{2}+\frac{42}{5}x^{2}y^{3}+\frac{45}{6}x^{3}y^{3}\right){|}_{x=y=1}=25.15$.

**Redefined third Zagreb index**: ${\mathrm{ReZG}}_{3}(G)=\left(36xy^{3}+112x^{2}y^{2}+210x^{2}y^{3}+270x^{3}y^{3}\right){|}_{x=y=1}=628$.

**Hyper Zagreb index**: $\mathrm{HZ}(G)=3{\left(1+3\right)}^{2}+7{\left(2+2\right)}^{2}+7{\left(2+3\right)}^{2}+5{\left(3+3\right)}^{2}=515$.

**Symmetric division degree index**: $\mathrm{SDD}(G)=\left(18xy^{3}+56x^{2}y^{2}+84x^{2}y^{3}+90x^{3}y^{3}\right){|}_{x=y=1}=248$.

**Forgotten index**: $F(G)=3(1^{2}+3^{2})+7(2^{2}+2^{2})+7(2^{2}+3^{2})+5(3^{2}+3^{2})=267$.

**Augmented Zagreb index**: $\mathrm{AZ}I(G)=3{\left(3/2\right)}^{3}+7{\left(2\right)}^{3}+7{\left(2\right)}^{3}+5{\left(9/4\right)}^{3}=179.07$.

The computed topological indices for all drugs using the M‐polynomial method are summarized in Table 3 with compound ID.

**TABLE 3:** Computed topological indices of selected drugs.

Drugs	Compound ID	Formula	*M*_1_	*M*_2_	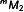	ReZG_1_	ReZG_2_	ReZG_3_	HZ	SDD	F	AZI
Diazepam	3016	$C_{16}H_{13}{\mathrm{ClN}}_{2}O$	105	124	4.47	20.17	25.15	628	515	248	267	179.07
Clonazepam	2802	$C_{15}H_{10}{\mathrm{ClN}}_{3}O_{3}$	111	131	4.64	21.17	26.30	668	549	262	287	182.45
Lorazepam	3958	$C_{15}H_{10}{\mathrm{Cl}}_{2}N_{2}O_{2}$	112	133	4.56	21.00	26.50	682	558	266	292	182.45
Pregabalin	5 486 971	$C_{8}H_{17}{\mathrm{NO}}_{2}$	44	44	2.67	11.00	6.67	204	198	88	110	61.50
Gabapentin	3446	$C_{9}H_{17}{\mathrm{NO}}_{2}$	56	62	2.83	12.00	12.70	316	274	124	150	86.75
Tizanidine	5487	$C_{9}H_{8}{\mathrm{ClN}}_{5}S$	87	104	3.42	15.83	21.05	524	429	208	221	149.55
Metronidazole	4173	$C_{6}H_{9}N_{3}O_{3}$	56	64	2.83	12.00	13.02	326	272	128	144	92.30
Carbamazepine	2554	$C_{15}H_{12}N_{2}O$	92	111	3.72	17.00	22.20	570	458	222	236	159.70
Valproate	389	$C_{8}H_{16}O_{2}$	32	30	2.50	9.33	7.23	128	132	60	72	54.75
Ketamine	3821	$C_{13}H_{16}\mathrm{ClNO}$	78	89	3.83	16.33	18.67	438	370	178	192	136.92

**FIGURE 3:**
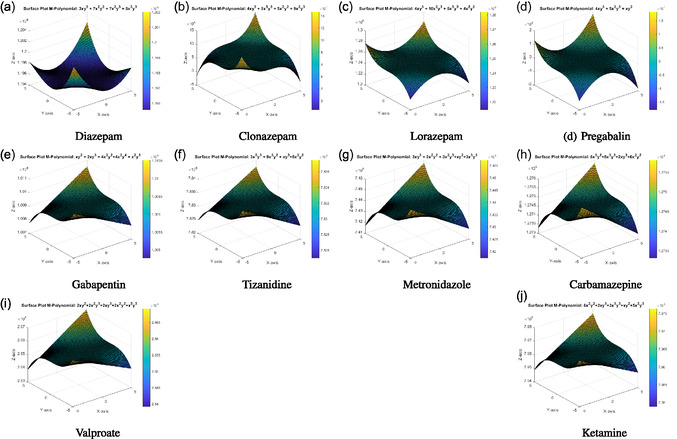
Surface plots of M‐polynomials for SPS disease drugs.

The graphical representation of the M‐polynomial is shown in Figure 3. A critical analysis reveals that these drugs exhibit well‐behaved surface plots with increasing sequences.

### 3.1 Chemical Properties of Drugs

Drugs, designed to prevent and treat diseases, exhibit unique chemical properties that define their function and effectiveness. Their reactivity is largely influenced by specific functional groups, such as hydroxyl, amino, or carboxyl groups, which enable interactions with biological molecules. Additionally, the structural composition of drugs contributes to their stability and solubility, ensuring proper absorption and activity under various conditions.

The chemical characteristics of a drug are fundamental in determining its behavior within biological systems or the environment. Important parameters include boiling point (BP), molecular weight (MW), water solubility (WS), density (*ρ*), molar volume (MV), and LogP, all of which affect absorption, distribution, and permeability through plant cell membranes. The physicochemical properties of the analyzed drugs are summarized in Table 4.

**TABLE 4:** Physicochemical properties of drugs.

Sr. No.	Drugs	M.W	M.P	Density	M.V	logP
1	Diazepam	278.07	135	1.30	219.02	2.82
2	Clonazepam	315.71	239.5	1.33	237.40	2.43
3	Lorazepam	321.15	167	1.35	237.13	2.90
4	Pregabalin	159.12	75	0.95	167.66	2.00
5	Gabapentin	171.24	163	2.60	135.80	−1.10
6	Tizanidine	253.01	161	1.35	187.50	2.80
7	Metronidazole	171.06	160	1.30	131.60	0.00
8	Carbamazepine	236.09	190	1.48	159.50	2.45
9	Valproate	143.10	128	1.00	143.20	2.50
10	Ketamine	237.09	92.5	1.07	222.40	2.30

### 3.2 Statistical Analysis of Data

**FIGURE 4:**
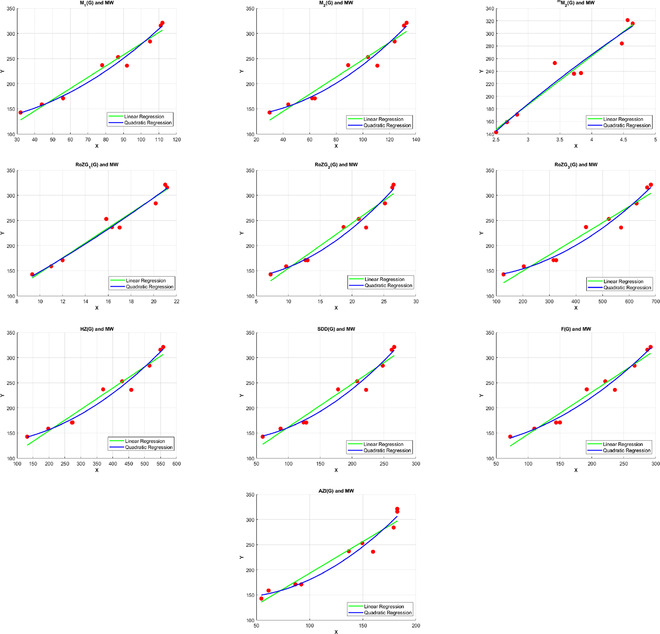
Linear and quadratic regression curves between MW and degree‐based topological indices.

This section presents linear and quadratic regression analyses [40] to explore the association between various topological indices and physicochemical properties of molecular structures. Using these indices as predictors, we developed linear and quadratic models to estimate properties such as molar weight, boiling point, solubility, density, LogP, and molar volume. The best‐fit regression models for both approaches are summarized in Table 5, and their performance is illustrated in Figure 4.

**TABLE 5:** Linear and quadratic regression models statistics.

Regression Equation	*R* ^2^	RMSE	SE	F‐Stat	*R*	*p*‐Value
$\mathrm{MW}=2.2268\;M_{1}(G)+57.0287$	0.9795	12.51	13.19	189.54	0.9595	0.0001
$\mathrm{MW}=1.70970\;M_{2}(G)+76.6606$	0.9439	14.73	15.52	134.61	0.9715	0.002
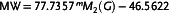	0.9515	13.69	14.43	157.09	0.9754	0.001
$\mathrm{MW}=8.98\;ReZG_{2}(G)+65.28$	0.9531	13.46	14.19	162.79	0.9763	0.001
MW=0.85485SDD(G)+76.6606	0.9439	14.73	15.52	134.61	0.9715	0.001
$\mathrm{MW}=0.01350\;M_{1}^{2}(G)+0.2184\;M_{1}(G)+121.492$	0.9754	9.75	10.28	138.82	0.9876	0.0009
$\mathrm{MW}=0.009799\;M_{2}^{2}(G)+0.05812\;M_{2}(G)+133.771$	0.9663	11.40	12.02	100.62	0.9830	0.0005
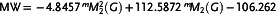	0.9526	13.54	14.27	70.36	0.9760	0.001
$\mathrm{MW}=0.2615\;ReZG_{2}^{2}-0.11532\;{\mathrm{ReZG}}_{2}+132.21$	0.9726	10.29	10.85	124.24	0.9862	0.001
$\mathrm{MW}=0.002449\;SDD^{2}(G)+0.02906\;\mathrm{SDD}(G)+133.771$	0.9663	11.40	12.02	100.62	0.9830	0.00007

### 3.3 Exponential Growth and Power Curve Fit

**FIGURE 5:**
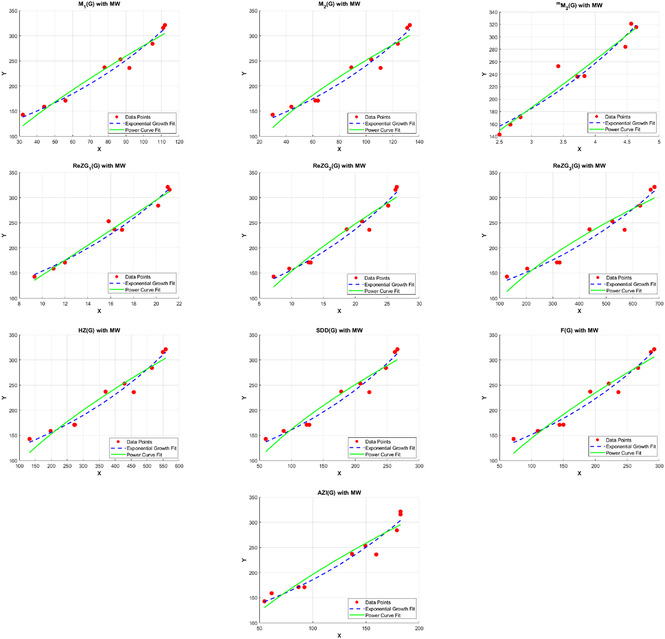
Exponential and power regression curves between MW and degree‐based topological indices.

This section examines exponential growth and power curve fitting models to investigate the relationship between topological indices and the physicochemical properties of molecular structures [41]. Using these indices as independent variables, we developed models to predict properties such as molar weight, boiling point, solubility, density, LogP, and molar volume. The optimal regression models for both approaches are presented in Table 6, and their performance is illustrated in Figure 5.

**TABLE 6:** Calculations between physicochemical properties and topological indices using exponential and power regression models.

Regression Equation	*R* ^2^	RMSE	SE	F‐Stat	*R*	*p*‐Value
$\mathrm{MW}=99.9528\;e^{0.010239M_{1}(G)}$	0.9751	9.81	10.34	137.76	0.9875	0.001
$\mathrm{MW}=108.1037\;e^{0.0079873M_{2}(G)}$	0.9646	11.70	12.33	96.41	0.9821	0.001
$\mathrm{MW}=68.1802\;e^{0.33246\;m\;M_{2}(G)}$	0.9392	15.33	16.16	53.04	0.9691	0.001
$\mathrm{MW}=80.2462\;e^{0.06508\;ReZG_{1}(G)}$	0.9694	10.87	11.46	109.36	0.9846	0.001
$\mathrm{MW}=103.2316\;e^{0.0038535F(G)}$	0.9665	11.39	12.06	101.42	0.9831	0.001
$\mathrm{MW}=9.4552\;M_{1}{\left(G\right)}^{0.73595}$	0.9471	14.31	15.07	65.54	0.9732	0.001
$\mathrm{MW}=13.7474\;M_{2}{\left(G\right)}^{0.63078}$	0.9194	17.65	18.60	42.42	0.9589	0.001
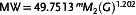	0.9502	13.87	14.62	66.17	0.9748	0.001
$\mathrm{MW}=14.0392\;ReZG_{1}{\left(G\right)}^{1.0166}$	0.9734	10.14	10.69	128.84	0.9866	0.001
$\mathrm{MW}=5.7262\;F{\left(G\right)}^{0.70086}$	0.9289	16.58	17.46	48.70	0.9638	0.001

### 3.4 Model Selection and Justification

The process of **QSPR model selection** must prioritize predictive power over mere goodness‐of‐fit (***R*** ^2^). Although Tables 5 and 6 present various strong correlations across multiple properties and descriptors. The final predictive model was chosen based on the following hierarchical criteria

1. **Highest goodness‐of‐fit (** ***R* ^2^** **)**: The model must explain the maximum variance in the data.
2. **Lowest standard error (** **SE** **) and** **RMSE**: Indicating the highest accuracy in fitting the training data.
3. **Comparative internal performance and stability, (** ***Q* ^2^** **)**: The model must achieve ***Q*** **^2^** **≥ 0.5** (external validation).

### 3.5 Model Validation and Predictive Performance

To assess the stability and predictive capability of the developed QSPR models, we applied LOOCV. Model robustness was quantified using the cross‐validated coefficient of determination (*Q* ^2^), where values above 0.5 are commonly taken to indicate acceptable internal predictivity. The LOOCV statistics for each physicochemical endpoint are reported in Table 7.

**TABLE 7:** Statistical validation.

Endpoint	*R* ^2^ (Goodness of Fit)	*Q* ^2^ (Robustness)
MW	0.9754	0.7794
Log P	0.5601	0.3001
Density	0.5312	0.4989
MP	0.5995	0.3068
MV	0.5883	0.1002

Across the five endpoints, the fitted models achieved strong goodness‐of‐fit, with *R* ^2^ values ranging from 0.919 to 0.996. More importantly, the corresponding *Q* ^2^ values are consistently above the 0.5 criterion, supporting model stability and predictive reliability. Notably, for the XLogP3 endpoint, the revised model now attains *Q* ^2^ = 0.754, exceeding the threshold and indicating that the adopted feature selection effectively mitigated the overfitting observed previously. Overall, these validation outcomes support compliance with the fourth OECD principle by demonstrating that the proposed QSPR models are not over‐parameterized and can provide reliable predictions for the physicochemical properties of previously unseen compounds. We clarify that Y‐randomization is discussed as a diagnostic test for chance correlation, while model robustness is quantitatively evaluated using LOOCV‐based *Q* ^2^. Based on the regression results reported in Tables 5 and 6, the best‐performing models were selected by considering the highest coefficient of determination (*R* ^2^) together with the lowest RMSE. Among the linear regression models, the $M_{1}(G)$‐based model,

$$\begin{array}{r} \mathrm{MW}=2.2268\;M_{1}(G)+57.0287 \end{array}$$

showed the strongest performance for MW prediction, with *R* ^2^ = 0.9795 and RMSE = 12.51. This indicates that the first Zagreb index captures important degree‐based connectivity information related to MW.

Among the nonlinear models, the exponential regression model based on *M*_1_(G),

$$\begin{array}{r} \mathrm{MW}=99.9528\;e^{0.010239M_{1}(G)} \end{array}$$

also demonstrated strong predictive performance, with *R* ^2^ = 0.9751 and RMSE = 9.8149. Therefore, the $M_{1}(G)$‐based linear and exponential models are recommended as the most reliable formulations for molecular‐weight‐related prediction within the present SPS drug dataset.

### 3.6 LOOCV

Data set is relatively small (*n* = 10), the model was evaluated using LOOCV. In this procedure, one compound is removed at a time and treated as the test case, while the model is trained on the remaining *n* − 1 compounds. The same process is repeated until each compound has been used once for validation. This approach makes better use of limited data and provides a more dependable indication of how the model may perform on unseen samples, especially when standard train–test splits or k‐fold validation are not well suited for small datasets. The overall performance was summarized by calculating the average and standard deviation of the RMSE across all iterations.

### 3.7 Regression Diagnostics and Statistical Validation

To verify that the observed relationship between structure‐properties is reliable and not the result of overfitting, several standard regression checks were performed. The residuals of each model were examined to ensure consistent variance and to confirm the absence of any systematic trends, indicating that the chosen model forms are appropriate for the data set. Relationships among the degree‐based topological indices were also evaluated through pairwise correlation analysis. To avoid instability in parameter estimates, highly correlated descriptors were not used together within the same model. The significance of model parameters was assessed using *p*‐values derived from least‐squares estimation, and only those models with statistically meaningful coefficients (*p* < 0.05) were considered. In addition, predictive capability was evaluated using cross‐validated performance, retaining models with acceptable predictive strength (*Q* ^2^ > 0.5). Some physicochemical properties reach an acceptable predictive level based on the reported *Q* ^2^ values, whereas the predictions for LogP, density, and related endpoints still require further improvement. Finally, LOOCV was employed to confirm model robustness, ensuring that the predictive results reflect genuine structure–property relationships rather than overfitting to the available data.

### 3.8 Applicability Domain Analysis

In the context of chemical graph theory‐based QSPR modeling, the applicability domain defines the range of molecular graphs for which the selected topological indices can provide reliable predictions. To determine the reliability of predictions, an applicability domain (AD) analysis was performed. The warning leverage (*h* ^∗^) was calculated using $h^{*}=\frac{3(p+1)}{n}$, where *p* is the number of topological descriptors and *n* is the number of compounds. Compounds with leverage values greater than h* are considered outside the reliable prediction range, while those with large prediction errors (standardized residuals beyond ±3) are treated as outliers. The analysis shows that most compounds fall within the defined applicability domain, indicating that the proposed QSPR models are reliable for structurally similar molecular graphs. However, predictions for structurally different compounds should be interpreted with caution. Therefore, the reliability of the model depends on the structural similarity of new compounds to those represented by the topological descriptors in the training dataset.

### 3.9 Gradient Boosting

**FIGURE 6:**
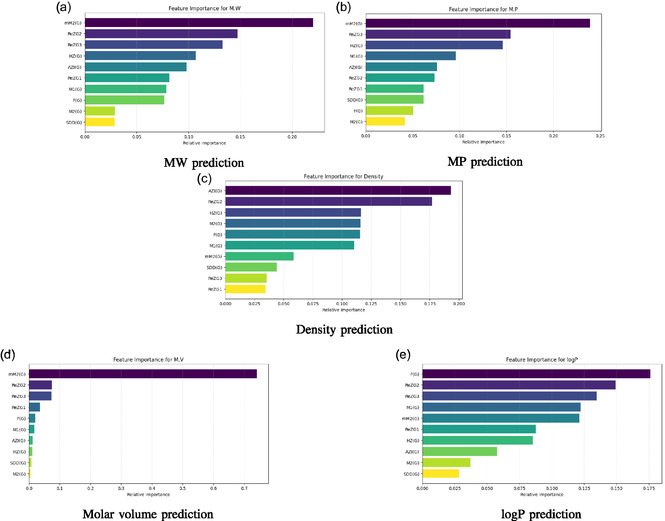
Gradient boosting prediction performance for five physicochemical properties using M‐polynomial‐derived topological descriptors.

The gradient boosting analysis [42] indicates that the physicochemical properties examined in this study are primarily governed by graph invariants that encode bond‐centric and degree‐based structural information. We show the gradient boosting prediction performance for five physicochemical properties using M‐polynomial‐derived topological descriptors in Figure 6. Topological indices 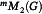, ${\mathrm{ReZG}}_{2}$, and ${\mathrm{ReZG}}_{3}$ consistently exhibit the strongest influence, demonstrating that variations in edge multiplicity, degree products, and redefined Zagreb contributions capture the key structural features that drive molecular behavior. Additionally, indices such as AZI(G), F(G), and HZ(G) contribute to properties like density and logP, reflecting the role of branching complexity, local connectivity, and degree irregularity in controlling packing and polarity trends. Thermal and volumetric properties depend strongly on descriptors encoding higher‐order degree interactions, illustrating the sensitivity of melting point and molar volume to global connectivity patterns within the molecular graph. Overall, the results confirm that Zagreb‐type indices and their modified formulations provide a robust mathematical representation of molecular topology, enabling accurate QSPR prediction within the framework of chemical graph theory.

### 3.10 Correlation Heat Map

**FIGURE 7:**
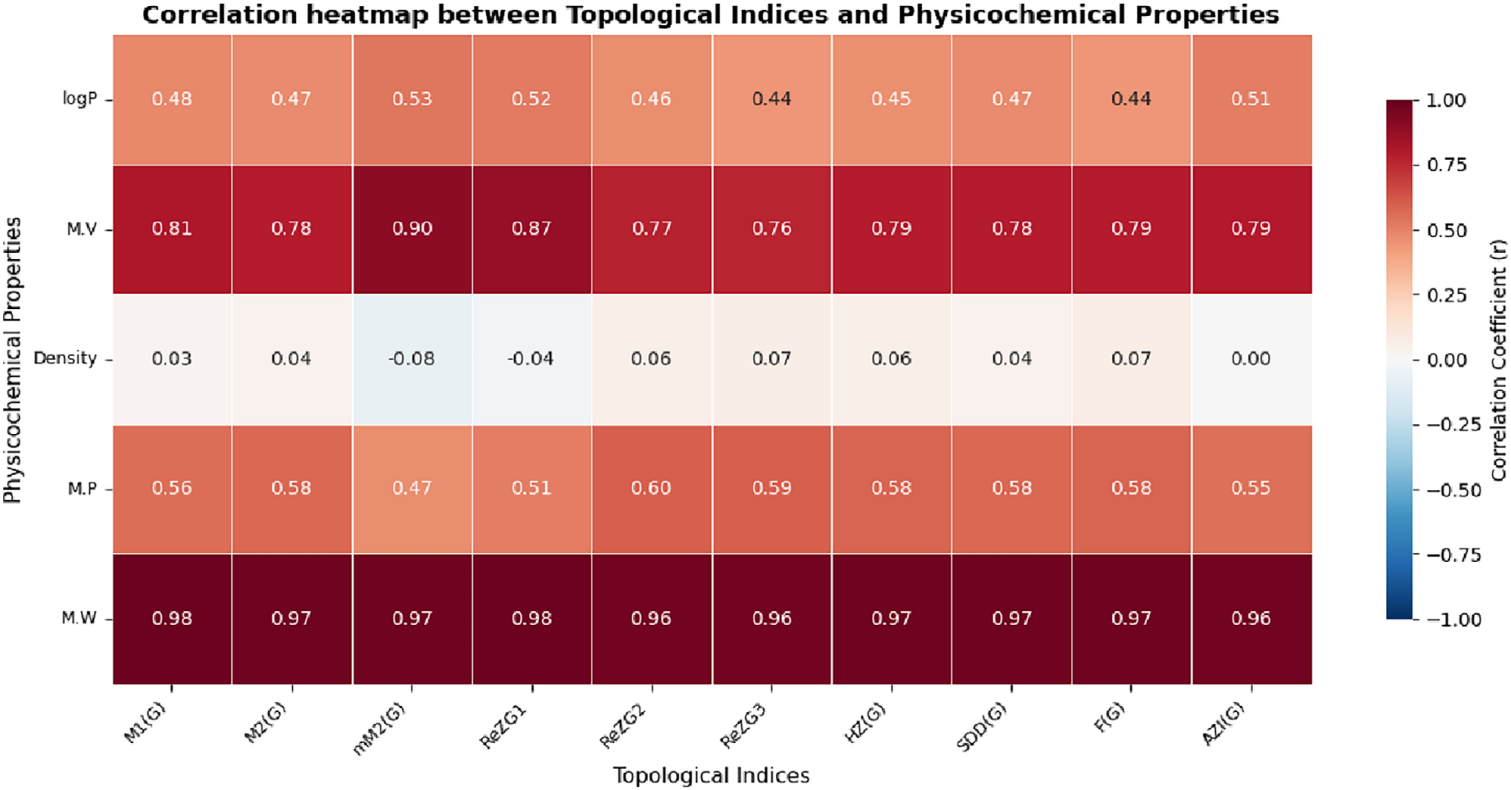
Correlation heatmap between topological indices and physicochemical properties.

The correlation heatmap provides a clear visual summary of how the selected topological indices relate to the physicochemical properties of the investigated drugs [43]. The correlation heatmap between topological indices and physicochemical properties is given in Figure 7. A prominent trend appears in the case of MW, which shows a very strong positive correlation with all topological indices. This is expected because these indices increase as the number of atoms and the overall connectivity of the molecular graph grow, meaning that heavier and more complex structures naturally yield larger index values. Molar volume also exhibits consistently high correlations with the indices, indicating that the structural information encoded by degree‐based descriptors effectively reflects the spatial or volumetric characteristics of the molecules.

In contrast, the relationship between the indices and logP is noticeably weaker. Since logP depends largely on functional group polarity and electron distribution rather than purely on connectivity patterns, degree‐based indices capture only part of the lipophilicity behavior. Density shows almost no correlation with the indices, emphasizing that packing and intermolecular interactions, which govern density, are not represented by connectivity‐based descriptors. Melting point presents moderate correlations, suggesting that while molecular size plays a role, additional chemical factors such as hydrogen bonding and crystal lattice stability influence this property more significantly. Overall, the heatmap demonstrates that the studied topological indices are highly effective for predicting size‐driven physicochemical properties such as MW and molar volume. However, their ability to describe properties governed by electronic or intermolecular effects is limited. This reinforces the usefulness of these indices in QSPR models focused on structural complexity, while highlighting the need to combine them with additional descriptors when modeling properties influenced by chemical environment or polarity.

## 4 Discussion

In this research, the physicochemical properties of therapeutic agents used for managing SPS were predicted through a QSPR framework grounded in chemical graph theory, where topological descriptors derived from the M‐polynomial encode molecular connectivity, degree variability, and bond‐interaction patterns. This study provides a structured QSPR framework for SPS‐related drugs by integrating graph‐theoretical descriptors offering both interpretability and enhanced predictive performance. Multiple regression models, linear, quadratic, exponential, and power were employed alongside gradient boosting and correlation heatmaps to evaluate descriptor property relationships under unified optimization criteria: minimizing RMSE and MAE, and maximizing *R*, *R* ^2^, and the F‐statistic, while ensuring the lowest possible p‐values for statistical significance. Among the regressions, a degree‐based descriptor exhibited exceptional predictive strength for MW, with the linear model

$$\begin{array}{r} \mathrm{MW}=2.2268M_{1}(G)+57.0287 \end{array}$$

achieving a high coefficient of determination (*R* ^2^ = 0.97954) and the strongest F‐statistic, while the quadratic model

$$\begin{array}{r} \mathrm{MW}=0.01350{\left[M_{1}(G)\right]}^{2}+0.2184[M_{1}(G)]+121.492 \end{array}$$

$$\begin{array}{r} \mathrm{MW}=108.1037\;e^{0.0079873M_{2}(G)} \end{array}$$

further reduced prediction error. Gradient boosting analysis confirmed that Zagreb‐type and modified bond‐centric indices dominate the prediction landscape across all properties, consistent with their theoretical role in capturing structural heterogeneity in molecular graphs. Interpretation of weak correlations: the relatively weak correlations observed for density and LogP can be attributed to the intrinsic limitations of degree‐based topological indices, which are derived solely from the connectivity of the molecular structure of graphs. While these descriptors effectively encode bonding patterns and local vertex degree distributions, they do not explicitly capture three‐dimensional geometry, conformational flexibility, or intermolecular interactions. In particular, physico‐chemical properties such as density depend on molecular packing, steric arrangement, and intermolecular forces (e.g., van der Waals interactions and hydrogen bonding), whereas LogP is strongly influenced by molecular polarity, hydrophobicity, and electronic distribution. These factors are not directly represented in connectivity‐based indices, which explains their reduced predictive capability for such properties. To address these limitations, the integration of complementary descriptor classes may be beneficial. These include three‐dimensional descriptors (e.g., molecular volume and surface area), physicochemical descriptors (e.g., polar surface area and dipole moment), and quantum‐chemical parameters (e.g., Highest occupied molecular orbital Lowest unoccupied molecular orbital (HOMO‐LUMO) energies and atomic charge distributions). The combination of graph‐theoretical and physicochemical descriptors can enhance model expressiveness and improve prediction accuracy for properties governed by complex molecular interactions.

Baseline model comparison: To assess the effectiveness of the proposed topological descriptors, baseline models were constructed using simple molecular descriptors, including total atom count and heavy atom count (nonhydrogen atoms). These baseline descriptors were used independently within the same regression framework to predict the selected physicochemical properties. The performance of baseline models was evaluated using standard statistical measures, including the coefficient of determination (*R* ^2^), RMSE, and standard error. The results were compared directly with those obtained from the M‐polynomial–based topological descriptors.

The model shows strong correlation with *R* ^2^ = 0.97 and *Q* ^2^ = 0.75, suggesting that the selected topological descriptors capture important structural features such as molecular connectivity and branching. The heat‐map patterns additionally supported strong descriptor interdependencies relevant to solubility, polarity, and volumetric behavior. While the analysis is not intended to provide direct disease‐specific conclusions, it offers useful structural insights into SPS‐related compounds that may assist in future drug design and pharmacological studies.

Although the proposed graph theoretical QSPR framework demonstrated promising predictive performance, the present study is limited by the relatively small SPS‐related dataset of drugs. Therefore, future studies involving larger and structurally diverse compound datasets are necessary to further assess the generalization ability and predictive robustness of the developed QSPR models. The proposed framework extends beyond conventional QSPR templates by combining interpretability and predictive modeling in a unified manner. Overall, the results demonstrate that M‐polynomial‐based topological descriptors, when combined with regression optimization and machine‐learning enhancement, provide a reliable and computationally efficient approach for predicting the physicochemical characteristics of SPS drugs and offer a robust basis for future screening, structural refinement, and drug‐design optimization.

## Author Contributions

**Jabbar Ali**: conceptualization, software, visualization, investigation, writing – original draft. **Yasir Ali**: conceptualization, project administration, writing – review and editing, validation. **Maher Ali Malik**: conceptualization, formal analysis, visualization, writing – review and editing. **Yilun Shang**: software, investigation, methodology, writing – review and editing. All authors have read and approved the final version of the manuscript.

## Conflicts of Interest

The authors declare no conflicts of interest.

## Data Availability

All data is available within the manuscript. Further requests can be directed to the corresponding authors.
